# Association of Halliwick-Based Aquatic Physical Activity with Health-Related Quality of Life in Children and Adolescents with Autism Spectrum Disorder: A Cross-Sectional Comparative Study

**DOI:** 10.3390/healthcare14193323

**Published:** 2026-10-06

**Authors:** Małgorzata Stefańska, Wiktoria Smętek, Joanna Kowalska, Agnieszka Ptak

**Affiliations:** 1 Faculty of Physiotherapy, Wroclaw University of Health and Sport Science, al. Paderewskiego 35, 51-612 Wrocław, Poland; joanna.kowalska@awf.wroc.pl (J.K.);; 2 Reh-Medica Marek Śmiech, ul. Odrzańska 7, 47-224 Kędzierzyn-Koźle, Poland; smetekw@gmail.com

**Keywords:** physical activity, Halliwick method, autism spectrum disorders, quality of life, KIDSSCREEN, aquatic physical activity

## Abstract

Background: Autism spectrum disorder (ASD) is a neurodevelopmental condition characterized by persistent deficits in social communication and interaction, accompanied by restricted and repetitive patterns of behavior, interests, or activities. Given the considerable heterogeneity in symptom severity and functional abilities, children with ASD require individualized educational and therapeutic interventions. Among the non-pharmacological approaches used to support this population, aquatic physical activity has emerged as a promising intervention. Material and Methods: The study included 45 children with ASD (ICD-11 code 6A02), comprising 19 girls and 26 boys, aged 8–17 years. Two groups were compared: Group G1, consisting of children and adolescents who had participated in Halliwick-based aquatic physical activity for at least 3 months, and Group G2, without aquatic activities. Health-related quality of life was assessed using the validated Polish version of the KIDSCREEN-52 questionnaire. Results: Children with ASD who participated in Halliwick-based aquatic physical activity (G1) had higher mean scores in all KIDSCREEN-52 domains. However, statistically significant differences were only found in the following domains: Psychological Well-Being, Moods and Emotions, Self-Perception, Autonomy, Financial Resources, and School Environment. Overall health-related quality of life, measured by the KIDSCREEN-10, compared with children who did not participate in aquatic activities. Conclusions: Halliwick-based aquatic physical activity was associated with better quality of life in children and adolescents with ASD, with higher scores observed according to sex, but further research is needed to confirm these findings.

## 1. Introduction

Autism spectrum disorder (ASD) is a neurodevelopmental disorder, characterized by impairments in social interaction and communication and repetitive restrictive behaviors [1]. As the prevalence of ASD across the world varies, the World Health Organization (WHO) estimates the global prevalence of ASD to be one person in 160 [2]. ASD persists throughout the lifespan [3]. The eleventh revision of the International Classification of Diseases (ICD-11) redefined the classification of autism by consolidating previous categories under ICD-10 into a single entity: Autism Spectrum Disorder (ICD-11 code 6A02). This change acknowledges autism’s spectrum nature and diverse clinical presentations. Core characteristics of ASD emerge in early development but may not be fully observable until social demands exceed the individual’s adaptive abilities. ICD-11 also recognizes variations in intellectual functioning and language development by introducing diagnostic specifiers, thereby offering a more nuanced framework for individualized diagnosis and treatment [2]. Individuals with ASD often experience difficulties with social interaction and communication, along with exhibiting restricted and repetitive behaviors [3].

Research indicates that children with ASD may experience motor difficulties including issues with postural control, gait abnormalities, and both gross and fine motor coordination. Consequently, these motor skill impairments can hinder their ability to engage in group physical activity programs [4,5]. These difficulties also include communication disorders, difficulties with social relationships, and specific, often repetitive patterns of behavior and interests [1]. Symptoms can vary in severity, which means that each child with ASD functions differently, requiring an individualized approach, both academic and therapeutic. Socially, children with autism spectrum disorders often exhibit difficulties interpreting emotions, building peer relationships, and maintaining eye contact. These problems impact their ability to adapt within a group, which can lead to isolation in a school setting [6]. Difficulties in daily functioning associated with ASD also impact a child’s overall quality of life. Research indicates that children with autism spectrum disorder often experience decreased mental well-being, limited physical activity, and difficulties in peer relationships [7]. Their self-esteem and sense of security may also be significantly lower than those of neurotypical children [7,8,9]. Therefore, it is important to monitor children’s quality of life using screening scales. Research shows that children are able to reliably express their opinions regarding quality of life when the questionnaire is appropriate for their developmental level [10]. One such scale that meets high methodological standards is KIDSCREEN. Despite this, there is little research on the quality of life of children with ASD. Early detection of a child’s deterioration in well-being can provide the basis for appropriate intervention and support. KIDSCREEN is a comprehensive instrument designed to assess health-related quality of life (HRQoL) in children and adolescents aged 8 to 18 years. It is available in both self-report and proxy-report formats, facilitating a thorough evaluation of subjective well-being. The KIDSCREEN-52 version encompasses ten distinct domains, which include physical and psychological well-being, social interactions with peers, school environment, experiences of bullying, and the availability of financial resources. Additionally, the KIDSCREEN-10 version provides a succinct measure for obtaining a singular global score, allowing for a streamlined assessment of overall quality of life. This multidimensional approach underscores the importance of understanding various factors influencing the well-being of youths [11,12]. The KIDSCREEN-10 has been demonstrated to possess psychometric adequacy as a comprehensive index for assessing global Health-Related Quality of Life (HRQoL) [11,12,13]. Among the many non-pharmacological methods used in the group of people with autism spectrum disorders, the following can be mentioned: aquatic physical activity (PA) [14]. Aquatic physical activity serves as a complementary intervention for children diagnosed with ASD, with the primary objective of enhancing motor skills while concurrently facilitating emotional regulation and promoting social participation [15,16]. The Hallwick method seems to be particularly useful in the case of children with ASD. The Halliwick method does not prescribe fixed swimming strokes, but (using the aquatic environment, primarily the phenomena of buoyancy and resistance) teaches body control and independence, from mental adaptation and balance control to progressive movement. Classes are conducted one-on-one (instructor and patient) in the form of games and fun [17]. Among the various methodologies employed in aquatic physical activity, the Halliwick approach is one of the interventions with documented use and evidence supporting its potential effectiveness in addressing various needs of this population [17,18]. Motor and aquatic skill improvements represent a consistently documented phenomenon within the literature pertaining to ASD. Programs centered around the Halliwick method and similar aquatic interventions have been shown to significantly enhance balance, water orientation, swimming proficiency, and overall gross motor performance among individuals with ASD [16,18,19]. Recent studies focusing on the Halliwick method have demonstrated notable enhancements in social performance, interpersonal relationships, and self-confidence among participants. Additionally, there are documented instances of a reduction in stereotypical behaviors. While a multitude of studies indicate positive changes in social, behavioral, and emotional outcomes, it is important to note that these improvements tend to be less consistent when compared to advancements observed in motor outcomes [16,19].

Evidence is limited regarding the association between regular Halliwick-based aquatic activity and self-reported multidimensional quality of life in children and adolescents with ASD.

Therefore, the aim of the study was to examine the association between aquatic physical activity using the Halliwick method and the health-related quality of life of children and adolescents with ASD.

We compared KIDSCREEN-52 and KIDSCREEN-10 scores between children and adolescents with ASD who had participated in weekly Halliwick-based aquatic physical activity for at least three months and peers who did not participate in organized aquatic activities.

In connection with the above, we hypothesized that aquatic physical activity participants would report higher quality of life scores, while recognizing that the cross-sectional, nonrandomized design permits assessment of associations rather than treatment effects.

## 2. Materials and Methods

### Study Group

The study was conducted at a swimming school in the Lower Silesia region, Poland, during the 2025–2026 period.

The study involved 45 children (19 girls and 26 boys) who met the inclusion criteria: age 8–17 years, diagnosis of autism spectrum disorder (ASD; ICD-11 code 6A02), no comorbidities (especially the presence of secondary neurological, orthopedic, or psychiatric disorders), consent from the child and parent or legal guardian to participate in the study, participation in physical activity in water using the Halliwick method at least once a week during 45 min sessions (Group G1). Exclusion criteria were also established: inability to independently complete the KIDSCREEN-52 questionnaire, and participation in physical activity in water using the Halliwick method for less than 3 months (Group G1). The study group size was estimated using the G*Power 3.1 program. To compare two independent samples, assuming a test power of 0.80, an effect size of 0.85 and *p* < 0.05, the estimated sample size should be 46 people. During the recruitment process, we were unable to achieve the planned sample size. The final study sample consisted of 45 participants rather than the intended 46.

All participants were familiarized with the purpose and principles of the study, the possibility of withdrawing at any stage without any consequences and agreed to participate. The study was conducted according to the guidelines of the Declaration of Helsinki and positive opinion from the Senate Commission for the Ethics of Scientific Research of the University Health and Sport Sciences in Wroclaw (no.: 50/2026; date: 30 January 2026). Preparatory organizational activities were undertaken before ethics approval; however, recruitment of participants and all procedures involving human subjects commenced only after the Ethics Committee approval had been granted. Before the study began, the children’s parents completed an informational survey. The most frequently reported limitations were social maladjustment (35% of the participants), learning difficulties (20% of the participants), low physical fitness (13% of the participants), emotional problems (7% of the participants), and self-harm (7% of the participants).

The children were divided into two groups:

- Group G1, *n* = 20, comprised children participating in aquatic physical activity using the Halliwick method;
- Group G2, *n* = 25, a comparison group, comprised children who did not participate in aquatic activities. Detailed characteristics of the participants are provided in Table 1.

## 3. Research Methods

The Polish version of the KIDSCREEN-52 questionnaire was used to examine the health-related quality of life of children and adolescents. Additionally, parents of the children studied completed an informational survey regarding sociodemographic data.

The KIDSCREEN-52 and the KIDSCREEN-10 questionnaires were used as the primary outcome measures. The KIDSCREEN-52 consists of 52 items grouped into ten dimensions of health-related quality of life: Physical Well-being, Psychological Well-being, Moods and Emotions, Self-Perception, Autonomy, Parent Relations and Home Life, Financial Resources, Social Support and Peers, School Environment, and Social Acceptance (Bullying). Each item is rated on a five-point Likert scale (0–4), reflecting either the frequency or intensity of a given experience during the previous week. Cronbach’s alpha coefficient for the ten sub-dimensions of the KIDSCREEN-52 ranged from 0.806 to 0.908 in the child-completed questionnaire [10].

Raw data were recoded into points 1–5 according to the instructions in the Polish version of the questionnaire [10] and on the website https://www.kidscreen.org/english/analysis/evaluation-by-hand/ (accessed 20 June 2026). Then, according to the protocol, sums were calculated for the 10 domains of the KIDSCREEN-52 questionnaire, and sums were calculated for the KIDSCREEN-10 questionnaire. KIDSCREEN-10 is designed as a unidimensional index, defined as a mental health index or a general health-related quality of life index. All obtained values were standardized to a scale of 0 to 100 and according to a ten-point scale with a mean of 50 and a standard deviation of 10 [10].

Group G1, consisting of children and adolescents, has participated in Halliwick-based in-aquatic physical activity at least once a week during 45 min sessions for at least three months. Classes were conducted one-on-one (by instructor and children) in the form of games and fun. The aquatic exercises were conducted individually based on the child’s abilities and skills. However, the foundation of the in-water activities is a logical, 10-point program consisting of the following steps:

1. Mental adjustment (building psychological comfort in a completely new environment).
2. Independence from others.
3. Transverse rotation control (e.g., learning to transition from lying on the back to standing).
4. Sagittal rotation control (e.g., learning to walk sideways).
5. Longitudinal rotation control (e.g., learning to transition from lying on the back to lying on the stomach).
6. Combined rotation control (e.g., learning to transition from lying on the back to lying on the stomach, turning, and standing).
7. Buoyancy.
8. Balance in stillness (e.g., repeating a sequence of movements combined with the ability to float and control breathing and movements of individual body parts).
9. Gliding in turbulence (practicing the ability to respond to sudden rotational movements).
10. Simple propulsion and basic swimming strokes.

By achieving subsequent points, the participant acquired more skills that allowed for greater activity and independence during the classes [20]. The classes were led by a certified Halliwick method therapist with many years of experience.

The participants in the comparison group did not participate in the Halliwick-based aquatic physical activity. They continued with their standard usual activities. Additionally, parents agreed to report to the researchers any new activities or exercises undertaken by their child.

## 4. Statistical Analysis

The distribution of all analyzed variables was assessed using the Shapiro–Wilk test and did not significantly deviate from a normal distribution. Descriptive statistics were calculated. The mean was used as a measure of central tendency, and the standard deviation for quantitative variables and counts and percentages for nominal variables were used as measures of dispersion. Levene’s test was used to verify homogeneity of variance. Depending on the results of the homogeneity of variance assessment, either Student’s t-test for independent samples (Welch’s *t*-test) or a test with independent variance estimation was used to assess the significance of differences between groups. For analyses involving variables with small subgroup sizes (*n* < 10), the Mann–Whitney U test was used to assess the significance of differences. Effect size was estimated using Cohen’s d. Differences in the levels of nominal variables were assessed using the chi-square test or Fisher’s exact test. A multiple linear regression model was calculated, maintaining the assumption of normality of residuals. Statistical analyses were performed in Statistica 14.1, PQStat 1.8.4, and online statistical calculators https://www.psychometrica.de/effect_size.html (accessed 9 June 2026) [21]. The level of significance was set at *p* < 0.05.

## 5. Results

Children with ASD participating in aquatic physical activity (G1) achieved higher scores in almost all domains of the KIDSCREEN-52 questionnaire and higher scores for overall quality of life, as assessed by the KIDSCREEN-10. Statistically significant differences were observed in the domains describing Psychological Well-Being, Moods and Emotions, Self-Perception, Autonomy, Financial Resources, School Environment, and the KIDSCREEN-10 total score. The largest difference between the G1 and G2 groups was observed in the Financial Resources domain. The highest scores on a 0–100 scale in both groups were observed in the Bullying, Parent Relations and Home Life, and Self-Perception domains. In the G1 group, t-scores for most subscales were above 50, except for Moods and Emotions and Peers and Social Support. The opposite pattern was observed in the G2 group, whose scores were mostly below 50 (Table 2).

When assessing the effect of participant age on KIDSCREEN scores, no significant differences were found between the younger and older groups. The exception was the Financial Resources domain, where older children scored significantly higher. In both groups, the highest scores on a 0–100 scale were achieved in Bullying, Parent Relations and Home Life, and Self-Perception (Table 3).

Statistically significant differences were observed between the estimated quality of life of girls and boys. Girls reported significantly higher health scores, a greater sense of autonomy, and higher overall quality of life scores. In the boys’ group, t-scores across all subscales and the estimated overall quality of life scores were lower than 50 (Table 4).

A multivariate regression model was developed with the dependent variable being the children’s overall quality of life score, estimated using the KIDSCREEN-10. Aquatic physical activity participation and sex were associated with KIDSCREEN-10 scores, but no significant effect of age was confirmed (Table 5). The obtained results prompted us to include an interaction term between sex and participation in aquatic physical activities. The subsequent analysis showed that only participation in aquatic physical activity was significantly associated with the children’s quality of life (Table 6).

Due to the significant association between sex and KIDSCREEN-10 scores, a comparison of the quality of life domains of children participating (G1) and those not participating in aquatic activities (G2) was performed separately for girls and boys. The G1 group of girls demonstrated significantly higher scores in 9/10 KIDSCREEN-52 domains. The G1 girls’ scores ranged from 76.65 to 96.12 on a 0–100 scale, while the G2 girls’ scores ranged from 69.44 to 82.78. Additionally, G1 girls had t-scores higher than 50 for most of the analyzed variables, while G2 girls’ scores were lower than 50 in most analyses (Table 7). The G1 boys’ group also demonstrated higher scores in all analyzed domains. However. statistical significance was only confirmed for Moods and Emotions and Self-Perception (Table 8).

## 6. Discussion

Autism spectrum disorder (ASD) is a lifelong neurodevelopmental condition that typically manifests during early childhood. The heterogeneity of the disorder means that symptoms can vary widely among individuals, necessitating a multidimensional approach to assessment and intervention [22]. Active participation in various life activities is a crucial element for children and adolescents with Autism Spectrum Disorder (ASD). According to the International Classification of Functioning, Disability, and Health (ICF), participation is defined as “involvement in life situations” [23]. This underscores the importance of fostering engagement in diverse social, educational, and recreational contexts to promote overall development and well-being among this population [8]. The International Classification of Functioning, Disability and Health (ICF) extends beyond the mere assessment of impairments in bodily functions and structures to emphasize the significance of participation as a critical determinant of health and well-being, particularly in pediatric and adolescent populations [8,24]. Research indicates that children and adolescents who are less actively engaged in social activities may experience lower self-esteem and evaluate their quality of life as less fulfilling across various domains, as measured by standardized quality-of-life instruments [25,26,27]. The findings align with the results of the present study. The construct of participation is further delineated into two dimensions: “attendance,” which denotes the mere presence in activities, and “engagement,” which addresses the qualitative aspects of involvement in those activities. For individuals diagnosed with ASD, challenges associated with body functions, particularly deficits in social communication and heightened sensory sensitivities, may significantly impede participation by constraining their ability to initiate, maintain, and adaptively engage in activities. These challenges are particularly pronounced in contexts necessitating social interaction or adjustment to multifaceted environments [3,7]. Nevertheless, research indicates the positive association and effectiveness of various physical activity interventions as methods for enhancing a range of abilities, including improving social skills and reducing behavioral problems in children with ASD [25]. Additionally, researchers emphasize the importance of designing individual intervention programs tailored to the different age groups and needs of children with ASD [7].

One of the therapeutic approaches utilized in the intervention process for children and adolescents with ASD is aquatic physical activity, as described by Halliwick. This method has been commonly employed to promote therapeutic outcomes in this population [17].

Generally, the studies suggest that participation in aquatic physical activity may have a positive connection with better functioning, including motor performance, social interaction, and emotional well-being [5]. Williams et al. also point out that poorer functioning, especially high levels of dependency, is associated with poorer quality of life. This poorer quality of life, in turn, can be partially explained by less frequent participation in social life [25]. Therefore, any intervention that improves psychophysical functioning may result in an improvement in the quality of life of children and adolescents with ASD.

In our study, children and adolescents engaged in supplementary aquatic activities demonstrated higher mean scores across all domains assessed by the KIDSCREEN-52 questionnaire compared to children who did not participate in such activities.

Güeita-Rodríguez et al. emphasize that the aquatic environment and water activities are suitable for increasing the subjective perception of quality of life in children with ASD [28]. Vodakova et al. also noted in their study the positive impact of a several-week aquatic activity program on the development of competences, which, in line with other findings, can be considered an indication of an increase in quality of life [29].

This finding suggests a positive correlation between participation in aquatic physical activities and well-being, highlighting the potential benefits of such interventions in promoting the health and quality of life among younger populations.

Due to the small number of studies on the relationship between aquatic physical activity and the quality of life of children with ASD, the findings of the present study can contribute to the growing body of information on aquatic physical activity as a complementary intervention for children with ASD.

Xing and Wu, in a systematic review from 2025, listed only seven studies (from 2010 to 2022) conducted in aquatic environments among various physical activities used in children and adolescents with ASD. This raises problems when comparing the obtained results and also highlights a research gap, which our study addresses [30].

Both the physical abilities and needs of children with ASD may change as they grow up, affecting their quality of life. Meanwhile, the findings of our research indicate that age was not significantly related to the outcomes of the quality of life of the studied children.

The results suggest the benefit of participation in aquatic physical activity regardless of age, which is in accordance with the literature [29]. This is indicated by comparisons of groups of children of different ages as well as the regression analysis performed. Also, van Heijst and Geurts in a meta-analysis of adults with ASD found that age did not affect quality of life [31]. However, this is a different study group.

The exception is the Financial Resources domain. This result is not surprising, as younger children are more financially dependent on their parents, a phenomenon that may change as they grow older and build independence, including financial independence. Thus, age-related differences in how participants perceive and interpret their financial resources should also be considered when interpreting this domain.

It should be noted that quality of life, including the Financial Resources domain, was self- reported by the participants themselves rather than assessed by their parents. Therefore, in our opinion, this domain does not necessarily reflect the actual financial situation of the family or directly explain the observed association with participation in aquatic physical activity. For children, participation in the aquatic physical activity classes included in the present study was free of charge and available specifically to individuals with ASD. Therefore, participation did not always involve additional financial costs for their families. For this reason, the findings related to the Financial Resources domain, as well as its potential association with participation in aquatic physical activity, should be interpreted with caution.

The results indicating differences in quality of life depending on sex in the entire study group of children seem interesting, confirmed by intergroup comparisons (girls vs. boys) and regression results. Girls reported a significantly better overall quality of life and significantly better results in domains related to physical and psychological well-being, autonomy, peers and social support, parent relations and home life compared to the group of boys. However, the regression model including the aquatic activity × sex interaction did not show this interaction to be statistically significant.

Further, but only exploratory in nature, analysis indicated a significantly higher quality of life in almost all domains among girls participating in aquatic physical activity in water compared to girls who were physically inactive in water. In the group of boys participating in aquatic physical activity, a significantly higher quality of life was noted only in the Moods and Emotions and Self-Perception domains, compared to boys who did not participate in such activities. This pattern requires confirmation in an adequately powered sample.

Previous research indicates that adolescent girls with ASD are at increased risk of developing anxiety and depression [32]. At the same time, girls, according to Hiller et al., have a greater need to build peer relationships and participate in group interactions more often than boys [29].

The authors are aware that the quality of life in the study group of children with ASD could have been influenced by various factors not considered in the study. Another limitation of the study was the single-center observational character of the study and small sample size, which prevented the development of a better regression model that takes into account more factors. Identifying other variables that may influence the overall quality of life score could have led to a better model fit. Furthermore, comparative analyses within specific subgroups (based on age, sex and participation in aquatic physical activity) were exploratory in nature. The limitations of the study are also differences between groups in participation in extracurricular therapy and socioeconomic confounding. This may influence the obtained research results, so the conclusions are very cautious and should not be generalized. Because of exclusion criteria, the results may not generalize to autistic children with intellectual disability, substantial communication difficulties, psychiatric comorbidity, or greater support needs.

The obtained results require confirmation in further studies on a larger, more homogeneous group, using randomization. At the same time, the quality of life assessment was conducted at a single point in time. We do not know the levels of quality of life in children with ASD before starting aquatic physical activity. Also, conducted according to Halliwick’s methodology, the aquatic physical activity was individualized, tailored to the child’s psychophysical abilities, and its intensity is difficult to determine.

Despite these limitations, the results are interesting and point to the future—adequately powered studies should examine whether sex modifies the association between aquatic activity and quality of life. Unfortunately, the scarcity of scientific publications on the quality of life of children with ASD, subjectively assessed by children rather than parents or guardians, as well as the use of different scales to assess children’s quality of life, makes it impossible to compare and contrast the obtained results. At the same time, this indicates the need for continued research on the quality of life of children and adolescents with ASD in the context of their physical activity in aquatic environments.

A logical next study would use a prospective multicenter design, baseline and follow-up health-related quality-of-life measurements, a standardized Halliwick dose, adherence monitoring, a prespecified primary outcome, measurement of socioeconomic and clinical confounders, and adequate sample size for a prespecified sex-interaction analysis.

## 7. Conclusions

Aquatic-activity participation and sex were associated with health-related quality of life scores in this sample.

The studied group of children with ASD participating in aquatic physical activity using the Halliwick method had a significantly higher quality of life (KIDSCREEN-10) compared to the group of children who did not participate in such activities.

Aquatic physical activity had a significant association with the health-related quality of life of the studied children with ASD.

Aquatic physical activity using the Halliwick method appears promising for children with ASD, in the context of their quality of life, but further studies are needed.

## Figures and Tables

**Table 1:** Characteristics of the study groups.

Aquatic Physical Activity (PA)						
		Yes, *n* = 20, G1		No, *n* = 25, G2		
		Mean	SD	Mean	SD	*p*
age (years)		11.75	2.17	11.68	2.67	0.9250
body height (cm)		148.60	15.25	149.32	15.02	0.8746
body mass (kg)		51.05	16.93	45.24	15.44	0.2360
		n	%	n	%	*χ*^2^/fisher’s *p*
sex	girls	7	35	12	48	0.4592
	boys	13	65	13	52	
school	public	4	20	4	16	0.1291
	integration	5	25	1	4	
	special	11	55	20	80	
extracurricular therapeutic activities	yes	15	75	9	34	0.0157 *
	no	5	25	16	64	
various forms of extracurricular therapeutic activities	sensory integration	11	55	8	32	0.0905
	speech therapy	2	10	3	12	
	behavioral therapy	3	15	1	4	

G1—group with PA; G2—comparison group without PA; * *p* < 0.05.

**Table 2:** Quality of life of children with ASD participating and not participating in aquatic physical activity.

Aquatic Physical Activity (PA)								
		Yes, *n* = 20, G1		No, *n* = 25, G2		Test T		Cohen’s d (95% CI)
KIDSCREEN-52:		Mean	SD	Mean	SD	t	*p*	
Physical Well-Being	S	72.20	17.29	66.56	15.23	1.16	0.2514	0.35 (0.24–0.94)
	t score	52.80	13.06	47.57	9.64	1.54	0.1297	0.46 (0.13–1.06)
Psychological Well-Being	S	81.34	14.36	68.40	17.21	2.69	0.0100 *	0.81 (0.20–1.42)
	t score	50.84	11.78	41.79	8.59	2.98	0.0048 *	0.89 (0.28–1.51)
Moods and Emotions	S	74.42	6.90	67.54	10.65	2.50	0.0164 *	0.75 (0.14–1.36)
	t score	42.84	3.11	39.86	4.34	2.58	0.0134 *	0.77 (0.17–1.38)
Self-Perception	S	86.80	12.32	76.64	14.50	2.49	0.0166 *	0.75 (0.14–1.36)
	t score	54.79	9.47	47.35	6.37	3.15	0.0030 *	0.95 (0.32–1.56)
Autonomy	S	80.00	19.64	66.56	18.18	2.38	0.0219 *	0.71 (0.11–1.32)
	t score	52.07	12.43	43.81	9.43	2.53	0.0150 *	0.76 (0.15–1.37)
Parent Relations and Home Life	S	86.01	12.31	80.13	15.82	1.36	0.1803	0.41 (−0.19–1.00)
	t score	52.36	10.33	47.78	9.45	1.55	0.1279	0.47 (−0.07–1.12)
Financial Resources	S	81.67	16.60	65.07	23.04	2.81	0.0075 *	0.81 (0.20–1.42)
	t score	51.42	7.64	45.04	10.14	2.33	0.0244 *	0.70 (0.09–1.31)
Peers and Social Support	S	72.34	22.80	60.26	21.12	1.84	0.0726	0.55 (−0.05–1.15)
	t score	47.91	14.24	40.68	13.40	1.75	0.0875	0.53 (−0.07–1.12)
School Environment	S	80.66	18.91	70.27	13.44	2.15	0.0370 *	0.65 (0.04–1.25)
	t score	54.85	12.23	47.88	7.42	2.36	0.0228 *	0.71 (0.10–1.31)
Bullying	S	91.33	9.45	86.14	12.30	1.55	0.1276	0.47 (−0.13–1.06)
	t score	49.26	9.53	44.73	9.25	1.61	0.1144	0.48 (−0.12–1.07)
KIDSCREEN-10	S	37.60	6.47	34.12	4.43	2.05	0.0483 *	0.64 (−0.06–1.14)
	t score	46.57	8.12	41.73	4.69	2.37	0.0249 *	0.75 (0.14–1.36)

G1—group with PA; G2—comparison group without PA; S—standardized values on a scale of 0–100; * *p* < 0.05.

**Table 3:** Quality of life of children with autism spectrum disorder depending on age.

		Age 8–11 *n* = 24		Age 12–17 *n* = 21		Test T		Cohen’s d (95% CI)
KIDSCREEN-52:		Mean	SD	Mean	SD	t	*p*	
Physical Well-Being	S	71.50	14.70	66.29	17.78	1.08	0.2876	0.33 (−0.26–0.92)
	t score	51.34	10.82	48.24	12.19	0.90	0.3717	0.27 (−0.32–0.86)
Psychological Well-Being	S	74.31	17.20	73.97	17.43	0.07	0.9476	0.02 (−0.57–0.61)
	t score	45.98	11.29	45.62	10.91	0.11	0.9155	0.03 (−0.55–0.62)
Moods and Emotions	S	72.38	10.03	68.57	9.16	1.32	0.1928	0.40 (−0.20–0.99)
	t score	42.00	4.25	40.25	3.77	1.45	0.1548	0.43 (−0.20–0.99)
Self-Perception	S	79.67	16.68	82.86	11.32	−0.74	0.4636	0.23 (−0.36–0.81)
	t score	50.12	9.35	51.26	7.96	−0.44	0.6648	0.13 (−0.45–0.72)
Autonomy	S	68.33	20.01	77.33	18.90	−1.54	0.1299	0.46 (−0.13–1.06)
	t score	44.94	11.09	50.38	11.56	−1.61	0.1148	0.48 (−0.11–1.07)
Parent Relations and Home Life	S	82.78	15.02	82.70	14.28	0.02	0.9857	0.01 (−0.58–0.59)
	t score	49.71	10.04	49.94	10.20	−0.07	0.9407	0.02 (−0.56–0.61)
Financial Resources	S	63.33	21.62	82.86	17.33	−3.31	0.0019 *	1.00 (0.38–1.63)
	t score	43.78	8.61	52.55	8.56	−3.42	0.0014 *	1.02 (0.40–1.64)
Peers and Social Support	S	60.83	20.72	71.11	23.61	−1.56	0.1269	0.47 (−0.13–1.06)
	t score	40.63	13.12	47.62	14.55	−1.70	0.0972	0.51 (−0.09–1.10)
School Environment	S	75.28	15.73	74.44	18.21	0.16	0.8701	0.05 (−0.54–0.64)
	t score	51.24	9.72	50.67	11.25	0.18	0.8567	0.05 (−0.53–0.64)
Bullying	S	88.34	9.05	88.57	13.68	−0.07	0.9458	0.02 (−0.57–0.61)
	t score	45.82	8.54	47.79	10.69	−0.69	0.4965	0.21 (−0.38–0.79)
KIDSCREEN-10	S	35.71	5.50	35.62	5.94	0.05	0.9585	0.02 (−0.57–0.60)
	t score	43.82	6.42	43.95	7.40	−0.06	0.9504	0.02 (−0.57–0.61)

S—standardized values on a scale of 0–100; * *p* < 0.05.

**Table 4:** Quality of life of children with ASD depending on sex.

		Girls *n* = 19		Boys *n* = 26		Test T		Cohen’s d (95% CI)
KIDSCREEN-52:		Mean	SD	Mean	SD	t	*p*	
Physical Well-Being	S	76.22	15.31	65.38	14.49	2.38	0.0217 *	0.73 (0.12–1.34)
	t score	55.47	11.81	46.78	9.41	2.71	0.0097 *	0.83 (0.21–1.45)
Psychological Well-Being	S	79.44	19.17	70.9	15.15	1.65	0.1065	0.56 (−0.04–1.16)
	t score	49.95	12.96	43.23	8.77	2.05	0.0464 *	0.63 (0.02–1.23)
Moods and Emotions	S	72.37	8.58	69.56	10.57	0.94	0.3549	0.29 (−0.31–0.88)
	t score	41.87	3.56	40.8	4.47	0.85	0.4026	0.26 (−0.33–0.85)
Self-Perception	S	84.89	13.41	79.23	14.67	1.3	0.2000	0.40 (−0.20–1.00)
	t score	52.56	8.49	49.68	8.72	1.09	0.2832	0.33 (−0.26–0.93)
Autonomy	S	79.56	20.57	67.38	18.37	2.06	0.0459 *	0.63 (0.03–1.24)
	t score	51.76	12.8	44.47	9.96	2.12	0.0396 *	0.65 (0.04–1.26)
Parent Relations and Home Life	S	88.15	16.69	79.49	11.95	2.01	0.0510	0.61 (0.01–1.22)
	t score	54.52	11.32	46.89	7.82	2.65	0.0113 *	0.81 (0.19–1.42)
Financial Resources	S	77.78	25.46	67.69	18.06	1.54	0.1312	0.47 (−0.13–1.07)
	t score	50.85	11.28	45.24	7.23	2.01	0.0504	0.61 (0.01–1.22)
Peers and Social Support	S	73.71	22.35	60.64	21.59	1.95	0.0584	0.60 (−0.01–1.20)
	t score	49.64	15.09	40.24	12.42	2.26	0.0292 *	0.69 (0.08–1.30)
School Environment	S	79.44	15.23	71.28	17.36	1.61	0.1150	0.49 (−0.11–1.10)
	t score	53.84	9.99	48.78	10.41	1.61	0.1150	0.49 (−0.11–1.10)
Bullying	S	87.78	8.93	89.23	12.93	−0.41	0.6830	0.13 (−0.47–0.72)
	t score	45.47	8.96	47.94	10	−0.84	0.4057	0.26 (−0.34–0.85)
KIDSCREEN-10	S	37.94	5.34	34.35	5.41	2.18	0.0349 *	0.67 (0.06–1.28)
	t score	46.64	6.96	42.25	6.19	2.2	0.0335 *	0.67 (0.07–1.28)

S—standardized values on a scale of 0–100; * *p* < 0.05.

**Table 5:** Multivariate regression model assessing the influence of PA, age and sex of children on the obtained KIDSCREEN-10 score.

Dependent Variable: KIDSCREEN-10	Coeff. b	SE b	−95% CI	+95% CI	t	*p*
PA: yes—1; no—0	4.30	1.54	1.19	7.41	2.79	0.0079 *
sex: girls—1; boys—2	−4.53	1.58	−7.73	−1.33	−2.86	0.0066 *
age (years)	−0.08	0.32	−0.72	0.56	−0.25	0.8023
Adjusted R^2^	0.20					
Standard Error of the Estimate (SEE)	5.04					
F	4.67					
*p*	0.0068 *					

PA—aquatic physical activity; * *p* < 0.05.

**Table 6:** Multivariate regression model assessing the influence of PA, age and sex and aquatic activity × sex interaction of children on the obtained KIDSCREEN-10 score.

Dependent Variable: KIDSCREEN-10	Coeff. b	SE b	−95% CI	+95% CI	t	*p*
PA: yes—1; no—0	13.07	5.31	2.33	23.81	2.46	0.0183 *
sex: girls—1; boys—2	−2.31	2.01	−6.38	1.77	−1.14	0.2590
age (years)	−0.14	0.31	−0.77	0.49	−0.46	0.6484
aquatic activity × sex interaction	−5.39	3.13	−11.72	0.94	−1.72	0.0930
Adjusted R^2^	0.24					
Standard Error of the Estimate (SEE)	4.93					
F	4.41					
*p*	0.0048 *					

PA—aquatic physical activity; * *p* < 0.05.

**Table 7:** Quality of life of girls with ASD participating and not participating in aquatic physical activity.

	PA—Aquatic Physical Activity							
		G1 Yes, *n* = 7		G2 No, *n* = 12		Test U MW		Cohen’s d (95% CI)
KIDSCREEN-52:		Mean	SD	Mean	SD	U	*p*	
Physical Well-Being	S	88.00	3.58	70.33	15.58	9.00	0.0131 *	1.39 (0.36–2.42)
	t score	65.62	6.27	50.39	10.65	9.00	0.0131 *	1.61 (0.55–2.68)
Psychological Well-Being	S	96.12	6.11	71.11	17.98	2.50	0.0020 *	1.67 (0.60–2.75)
	t score	63.37	7.98	43.24	9.13	2.50	0.0020 *	2.30 (1.12–3.49)
Moods and Emotions	S	76.65	4.19	70.23	9.53	16.00	0.0678	0.80 (−0.17–1.76)
	t score	43.77	1.99	40.92	3.85	16.00	0.0678	0.86 (−0.11–1.83)
Self-Perception	S	94.67	4.84	80.00	13.75	5.00	0.0043 *	1.29 (0.27–2.31)
	t score	60.44	7.67	48.62	5.82	5.00	0.0043 *	1.81 (0.71–2.91)
Autonomy	S	95.33	5.89	71.67	20.85	10.50	0.0192 *	1.38 (0.35–2.41)
	t score	62.20	7.65	46.54	11.72	10.50	0.0192 *	1.50 (0.45–2.54)
Parent Relations and Home Life	S	98.90	1.70	82.78	18.30	9.00	0.0131 *	1.01 (0.10–2.09)
	t score	63.42	3.79	50.07	11.26	9.00	0.0131 *	1.43 (0.39–2.47)
Financial Resources	S	94.44	5.02	69.44	27.63	18.00	0.1012	1.12 (0.12–2.11)
	t score	57.86	4.16	47.34	12.18	18.00	0.1012	1.04 (0.05–2.03)
Peers and Social Support	S	91.12	8.87	65.00	22.09	13.50	0.0394 *	1.41 (0.38–2.44)
	t score	59.43	10.55	44.75	14.93	13.50	0.0394 *	1.01 (0.09–2.08)
School Environment	S	91.65	9.38	73.33	14.01	8.50	0.0114 *	1.46 (0.42–2.50)
	t score	62.33	8.17	49.59	8.06	8.50	0.0114 *	1.57 (0.52–2.63)
Bullying	S	94.45	8.86	84.45	7.16	14.50	0.0492 *	1.28 (0.26–2.30)
	t score	52.65	9.69	41.88	6.27	14.50	0.0492 *	1.41 (0.37–2.44)
KIDSCREEN-10	S	43.00	2.10	35.42	4.60	1.00	0.0012 *	1.94 (0.82–3.06)
	t score	53.60	4.74	43.16	4.99	1.00	0.0012 *	2.13 (0.98–3.28)

G1—group with PA; G2—comparison group without PA; S—standardized values on a scale of 0–100; * *p* < 0.05.

**Table 8:** Quality of life of boys with ASD participating and not participating in aquatic physical activity.

		PA—Aquatic Physical Activity						
		G1 Yes, *n* = 13		G2 No, *n* = 13		Test T		Cohen’s d (95% CI)
KIDSCREEN-52:		Mean	SD	Mean	SD	t	*p*	
Physical Well-Being	S	67.69	14.56	63.08	14.62	0.81	0.4278	0.32 (−0.46–1.09)
	t score	48.59	10.54	44.97	8.15	0.98	0.3374	0.39 (−0.39–1.16)
Psychological Well-Being	S	75.91	11.94	65.89	16.78	1.75	0.0923	0.69 (−0.10–1.48)
	t score	46.01	8.74	40.46	8.21	1.67	0.1080	0.66 (−0.14–1.44)
Moods and Emotions	S	74.06	7.69	65.05	11.38	2.36	0.0265 *	0.93 (0.12–1.74)
	t score	42.71	3.45	38.88	4.68	2.37	0.0259 *	0.93 (0.12–1.74)
Self-Perception	S	84.92	12.35	73.54	15.01	2.11	0.0453 *	0.83 (0.03–1.63)
	t score	53.19	9.20	46.17	6.86	2.20	0.0375 *	0.86 (0.06–1.67)
Autonomy	S	72.92	20.60	61.85	14.57	1.58	0.1266	0.62 (−0.17–1.41)
	t score	47.65	12.12	41.29	6.14	1.69	0.1046	0.66 (−0.13–1.45)
Parent Relations and Home Life	S	81.28	10.50	77.69	13.42	0.76	0.4546	0.30 (−0.48–1.07)
	t score	48.12	8.47	45.66	7.22	0.80	0.4326	0.31 (−0.46–1.09)
Financial Resources	S	74.36	16.07	61.03	18.02	1.99	0.0580	0.78 (−0.02–1.58)
	t score	47.57	6.12	42.91	7.71	1.71	0.1010	0.67 (−0.12–1.46)
Peers and Social Support	S	65.39	22.81	55.88	20.05	1.13	0.2701	0.44 (−0.34–1.22)
	t score	43.55	13.22	36.93	11.08	1.38	0.1793	0.54 (−0.24–1.33)
School Environment	S	75.12	20.81	67.44	12.77	1.13	0.2676	0.45 (−0.33–1.22)
	t score	51.28	12.93	46.29	6.71	1.23	0.2293	0.48 (−0.30–1.26)
Bullying	S	90.76	9.64	87.70	15.82	0.60	0.5569	0.24 (−0.54–1.01)
	t score	48.54	9.39	47.35	10.93	0.30	0.7694	0.19 (−0.65–0.89)
KIDSCREEN-10	S	35.77	6.33	32.92	4.07	1.36	0.1852	0.54 (−0.25–1.32)
	t score	44.09	7.44	40.41	4.15	1.56	0.1319	0.61 (−0.18–1.40)

G1—group with PA; G2—comparison group without PA; S—standardized values on a scale of 0–100; * *p* < 0.05.

## Data Availability

The data that support the findings of this study are not openly available due to sensitivity reasons and are available from the corresponding author upon reasonable request. Data are located in controlled-access data storage.

## References

[1] American Psychiatric Association (2013). Diagnostic and Statistical Manual of Mental Disorders: DSM-5™.

[2] World Health Organization (2013). Autism Spectrum Disorders & Other Developmental Disorders: From Raising Awareness to Building Capacity.

[3] Lai M.C., Anagnostou E., Wiznitzer M., Allison C., Baron-Cohen S. (2020). Evidence-based support for autistic people across the lifespan: Maximising potential, minimising barriers, and optimising the person-environment fit. Lancet Neurol..

[4] Kruger G.R., Silveira J.R., Marques A.C. (2019). Motor skills of children with autism spectrum disorder. Rev. Bras. Cineantropometria Desempenho Hum..

[5] Dame C., Viellard M., Elissalde S.-N., Pergeline H., Grandgeorge P., Garie L.-A., Solla F., De Martino S., Avenel E., Salle-Collemiche X. (2024). Developmental Profile of Children with Autism Spectrum Disorder Versus Social Communication Disorder: A Pilot Study. Children.

[6] Bhat A.N., Boulton A.J., Tulsky D.S. (2022). A further study of relations between motor impairment and social communication, cognitive, language, functional impairments, and repetitive behavior severity in children with ASD using the SPARK study dataset. Autism Res..

[7] Tedla J.S., Asiri F., Reddy R.S., Sangadala D.R., Gular K., Kakaraparthi V.N. (2024). Assessing the quality of life in children with autism spectrum disorder: A cross-sectional study of contributing factors. Front. Psychiatry.

[8] Lin C.-Y., Kang L.-J., Liao H.-F., Cheong P.-L., Yen C.-F., Liou T.-H., Wu Y.-T., Yang P.-Y. (2026). Participation patterns and associated factors in children and adolescents with autism spectrum disorder: A nationwide population study. Res. Dev. Disabil..

[9] Dunlap W.P. (1994). Generalizing the common language effect size indicator to bivariate normal correlations. Psychol. Bull..

[10] Mazur J., Malkowska-Szkutnik A., Dzielska A., Tabak I. (2008). Polska Wersja Kwestionariuszy do Badania Jakości Życia Związanej ze Zdrowiem Dzieci i Młodzieży (KIDSCREEN).

[11] Befus E.G., Helseth S., Mølland E., Westergren T., Fegran L., Haraldstad K. (2023). Use of KIDSCREEN health-related quality of life instruments in the general population of children and adolescents: A scoping review. Health Qual. Life Outcomes.

[12] Ravens-Sieberer U., Erhart M., Rajmil L., Herdman M., Auquier P., Bruil J., Power M., Duer W., Abel T., Czemy L. (2010). Reliability, construct and criterion validity of the KIDSCREEN-10 score: A short measure for children and adolescents’ well-being and health-related quality of life. Qual. Life Res..

[13] Coales C., Heaney N., Ricketts J., E Dockrell J., Lindsay G., Palikara O., Charman T. (2019). Health-related quality of life in children with autism spectrum disorders and children with developmental language disorders. Autism Dev. Lang. Impair..

[14] Ogonowska-Slodownik A., Jakobowicz O., Alexander L., Marinho-Buzelli A.R., Devion C., Morgulec-Adamowicz N. (2024). Aquatic Therapy in Children and Adolescents with Disabilities: A Scoping Review. Children.

[15] Hooft P.v.T., Moeijes J., Hartman C., van Busschbach J., Hartman E. (2026). Aquatic Interventions to Improve Motor and Social Functioning in Children with ASD: A Systematic Review. Rev. J. Autism Dev. Disord..

[16] Mortimer R., Privopoulos M., Kumar S. (2014). The effectiveness of hydrotherapy in the treatment of social and behavioral aspects of children with autism spectrum disorders: A systematic review. J. Multidiscip. Healthc..

[17] Kokaridas D., Lambeck J. (2015). The Halliwick Concept: Toward A Collaborative Aquatic Approach. Inq. Phys. Educ. Sport.

[18] Shariat A., Najafabadi M.G., dos Santos I.K., Anastasio A.T., Milajerdi H.R., Hassanzadeh G., Nouri E. (2024). The Effectiveness of Aquatic Therapy on Motor and Social Skill as Well as Executive Function in Children with Neurodevelopmental Disorder: A Systematic Review and Meta-analysis. Arch. Phys. Med. Rehabil..

[19] Marzouki H., Soussi B., Selmi O., Hajji Y., Marsigliante S., Bouhlel E., Muscella A., Weiss K., Knechtle B. (2022). Effects of Aquatic Training in Children with Autism Spectrum Disorder. Biology.

[20] Weber-Nowakowska K., Zyzniewska-Banaszk E., Gebska M. (2011). New methods in physiotherapy. The Halliwick concept as a form of rehabilitation in water. Ann. Acad. Medicae Stetin..

[21] Lenhard W., Lenhard A. (2017). Computation of Effect Sizes.

[22] Chaput J.-P., Willumsen J., Bull F., Chou R., Ekelund U., Firth J., Jago R., Ortega F.B., Katzmarzyk P.T. (2020). 2020 WHO guidelines on physical activity and sedentary behaviour for children and adolescents aged 5–17 years: Summary of the evidence. Int. J. Behav. Nutr. Phys. Act..

[23] World Health Organization ICD-11 for Mortality and Morbidity Statistics. https://icd.who.int/browse/2022-02/mms/en.

[24] Egilson S.T., Jakobsdóttir G., Ólafsson K., Leósdóttir T. (2017). Community participation and environment of children with and without autism spectrum disorder: Parent perspectives. Scand. J. Occup. Ther..

[25] Williams K., Jacoby P., Whitehouse A., Kim R., Epstein A., Murphy N., Reid S., Leonard H., Reddihough D., Downs J. (2021). Functioning, participation, and quality of life in children with intellectual disability: An observational study. Dev. Med. Child Neurol..

[26] Kong C., Chen A., Ludyga S., Herold F., Healy S., Zhao M., Taylor A., Müller N.G., Kramer A.F., Chen S. (2023). Associations between meeting 24-hour movement guidelines and quality of life among children and adolescents with autism spectrum disorder. J. Sport Health Sci..

[27] Krieger B., Piškur B., Beurskens A.J.H.M., Moser A. (2024). Parents’ perceptions: Participation patterns and desires for change for children and adolescents with autism spectrum disorder-A descriptive population-based study from Switzerland. Child Care Health Dev..

[28] Güeita-Rodríguez J., Ogonowska-Slodownik A., Morgulec-Adamowicz N., Martín-Prades M.L., Cuenca-Zaldívar J.N., Palacios-Ceña D. (2021). Effects of Aquatic Therapy for Children with Autism Spectrum Disorder on Social Competence and Quality of Life: A Mixed Methods Study. Int. J. Environ. Res. Public Health.

[29] Vodakova E., Chatziioannou D., Jesina O., Kudlacek M. (2022). The Effect of Halliwick Method on Aquatic Skills of Children with Autism Spectrum Disorder. Int. J. Environ. Res. Public Health.

[30] Xing Y., Wu X. (2025). Effects of Motor Skills and Physical Activity Interventions on Motor Development in Children with Autism Spectrum Disorder: A Systematic Review. Healthcare.

[31] van Heijst B.F.C., Geurts H.M. (2015). Quality of life in autism across the lifespan: A meta-analysis. Autism.

[32] Hiller R.M., Young R.L., Weber N. (2014). Sex differences in autism spectrum disorder based on DSM-5 criteria: Evidence from clinician and teacher reporting. J. Abnorm. Child Psychol..

